# Global burden of lower extremity peripheral arterial disease attributable to smoking from 1990 to 2021: A secondary dataset analysis of the Global Burden of Disease Study 2021

**DOI:** 10.18332/tid/219768

**Published:** 2026-05-07

**Authors:** Ze Chen, Ke Yuan, Chunxiao Wan

**Affiliations:** 1 Department of Physical Rehabilitation and Medicine, Tianjin Medical University General Hospital, Tianjin, China; 2 College of Rehabilitation Medicine, Tianjin Medical University, Tianjin, China

**Keywords:** lower extremity peripheral arterial disease, smoking, Global Burden of Disease, age-period-cohort analysis

## Abstract

**INTRODUCTION:**

Smoking is the primary modifiable risk factor for lower extremity peripheral arterial disease (LEPAD). This study aimed to assess the global spatiotemporal trends of LEPAD burden attributable to smoking and disentangle the underlying drivers.

**METHODS:**

This study is a secondary analysis of publicly available data from the Global Burden of Disease (GBD) 2021 study. We analyzed mortality and disability-adjusted life years (DALYs) across 204 countries and territories from 1990 to 2021. Trends were evaluated using estimated annual percentage change (EAPC), joinpoint regression, and age-period-cohort (APC) models.

**RESULTS:**

In 2021, the global number of deaths and disability-adjusted life years (DALYs) due to LEPAD attributable to smoking reached 14130.84 (95% UI: 10300.51–18587.81) and 439653.32 (95% UI: 306683.31–602207.38), respectively, representing increases of 32.25% and 41.01% since 1990, largely driven by population aging. Conversely, the age-standardized mortality rate (ASMR) decreased by 47.25% to 0.16 per 100000, and the age-standardized DALY rate (ASDR) decreased by 40.94% to 5.01 per 100000. Males bore approximately 3.5 times the mortality burden of females (10986.02 vs 3144.82 deaths), and the highest rates were observed in Eastern Europe and Central Asia. EAPC analysis showed a negative correlation with the sociodemographic index (SDI), indicating rising trends in some low SDI regions. The APC analysis revealed that while risk escalated exponentially with age, favorable period and cohort effects drove the decline in rates.

**CONCLUSIONS:**

Although age-standardized rates have declined globally due to tobacco control and medical advancements, the absolute burden of smoking-attributable LEPAD continues to rise. Targeted interventions focusing on high-risk regions and aging populations should be considered to mitigate this growing challenge.

## INTRODUCTION

Lower extremity peripheral arterial disease (LEPAD) is a major manifestation of systemic atherosclerosis and a significant contributor to cardiovascular morbidity and mortality worldwide^1,2^. With the rapid aging of the global population, the prevalence of LEPAD has risen steadily, affecting more than 200 million people globally^3^. LEPAD is associated with severe clinical outcomes, ranging from intermittent claudication to critical limb ischemia requiring amputation, which substantially impairs quality of life and imposes a heavy socio-economic burden^4^. Identifying and managing key risk factors for LEPAD is, therefore, critical for developing effective public health prevention strategies.

Among various cardiovascular risk factors, smoking is recognized as the single most important modifiable risk factor for the development and progression of LEPAD^5^. Epidemiological evidence suggests that the association between smoking and LEPAD is stronger than that for coronary heart disease or stroke^6^. Harmful constituents in tobacco smoke induce endothelial dysfunction, promote inflammation, and accelerate thrombosis, thereby exacerbating the atherosclerotic process^7^. Although substantial global efforts have been made in tobacco control over the past few decades, smoking prevalence remains high in many low- and middle-income countries^8^. Furthermore, demographic shifts may be altering the epidemiological profile of smoking-attributable disease burden^8^.

While previous studies based on the Global Burden of Disease (GBD) study have described the overall burden of peripheral arterial disease^3^, comprehensive analyses specifically focusing on the long-term spatiotemporal trends of LEPAD burden attributable to smoking remain scarce. Moreover, trends in disease mortality and morbidity are driven by a complex interplay of age (biological risk), period (macro-environmental factors affecting all age groups), and cohort effects (lifestyle or exposure patterns specific to generations)^9^. However, traditional descriptive analyses may not adequately disentangle the independent effects of age, period, and cohort on disease trends, potentially leading to incomplete or misleading interpretations of the observed patterns.

Therefore, using the latest data from the Global Burden of Disease Study 2021 (GBD 2021), this study aims to assess global, regional, and national trends in mortality and disability-adjusted life years (DALYs) attributable to smoking for LEPAD from 1990 to 2021. Additionally, an age-period-cohort model was employed to independently estimate the specific effects of age, period, and birth cohort on these trends.

## METHODS

### Data source

This study is a secondary analysis of publicly available data from the Global Burden of Disease (GBD) 2021 study, conducted between 1990 and 2021. Coordinated by the Institute for Health Metrics and Evaluation (IHME) at the University of Washington, the GBD 2021 is a comprehensive observational epidemiological study that quantifies health loss from hundreds of diseases, injuries, and risk factors^10,11^. The GBD study integrates multiple data sources, including censuses, household surveys, civil registration and vital statistics, disease registries, and scientific literature, using statistical modeling techniques to generate consistent estimates. The GBD 2021 provides estimates for 204 countries and territories, which are geographically grouped into 21 GBD regions and 7 super-regions, spanning the 32-year period from 1990 to 2021 ^11^. This study extracted annual data on the burden of lower extremity peripheral arterial disease (LEPAD) attributable to smoking using the Global Health Data Exchange (GHDx) query tool^12^.

This study utilized secondary, aggregated data that are publicly available and de-identified. As the analysis did not involve direct interaction with human subjects or access to identifiable personal information, the requirement for ethical approval and informed consent was waived by the institutional review board (IRB).

### Case definition and risk factors

In GBD 2021, the reference case definition for lower extremity peripheral arterial disease (LEPAD) was defined as having an ankle-brachial index (ABI) ≤0.9. Clinically, symptomatic LEPAD was defined as intermittent claudication (leg pain on exertion) among individuals meeting the ABI threshold. To capture the comprehensive burden using administrative and clinical data, the GBD study mapped hospital inpatient, outpatient, and insurance claims data to LEPAD using International Classification of Diseases (ICD) codes. According to the GBD 2021 methodology, the included ICD-10 codes primarily comprised I70.2–I70.29 (atherosclerosis of native arteries of the extremities, covering intermittent claudication, rest pain, ulceration, and gangrene) and I73 (other peripheral vascular diseases) along with its subcategories (e.g. I73.1, I73.8, I73.9). Corresponding ICD-9 codes (e.g. 440.20–440.24, 443) were also utilized. Data sources utilizing alternative case definitions (e.g. claims data without direct ABI measurement) were adjusted to the reference definition using the Meta-Regression-Bayesian, Regularized, Trimmed (MR-BRT) tool to correct for bias. The overall prevalence was split into asymptomatic and symptomatic (intermittent claudication) categories, with specific disability weights applied to estimate years lived with disability (YLDs)^11^.

In this study, ‘smoking’ encompasses the use of any smoked tobacco product (including manufactured cigarettes, hand-rolled cigarettes, cigars, pipes, hookah or water pipe, bidis, kreteks, and other locally produced smoked tobacco products). Within the GBD 2021 analytical structure, smoking exposure is stratified into two distinct groups. Current smokers encompass individuals who, at the time of assessment, reported active consumption of any combustible tobacco product, regardless of whether use occurred daily or intermittently. Former smokers refer to those who had completely ceased the use of all combustible tobacco products for a continuous period of no less than six months prior to the survey. To capture cumulative exposure, continuous indicators such as ‘cigarettes per smoker per day’ and ‘pack-years’ were modeled using Spatio-Temporal Gaussian Process Regression (ST-GPR) based on nationally representative household surveys. The burden of LEPAD attributable to smoking was quantified using the population attributable fraction (PAF), which represents the proportional reduction in risk if the population exposure shifted to the Theoretical Minimum Risk Exposure Level (TMREL) of zero (never smoking). The detailed methodology for calculating PAFs, based on the comparison between the actual exposure distribution and the counterfactual distribution at TMREL, has been described extensively elsewhere^10^. Notably, GBD 2021 implemented a significant methodological advancement by estimating relative risks (RR) using the MR-BRT tool. Specifically for cardiovascular outcomes including LEPAD, age-specific dose-response risk curves were generated to account for the attenuation of excess risk with increasing age, thereby providing more precise estimates compared to previous GBD cycles^8,10,13^.

### Burden metrics and sociodemographic index

The burden of LEPAD attributable to smoking was quantified using two primary metrics: absolute number of deaths and disability-adjusted life years (DALYs). DALYs serve as a comprehensive measure of health loss, calculated as the sum of years of life lost (YLLs) due to premature mortality and years lived with disability (YLDs)^11^. To account for variations in population age structures across different locations and time periods, this study computed age-standardized rates (ASRs) per 100000 population using the direct standardization method based on the GBD world standard population^14^. To assess the relationship between disease burden and development status, this study utilized the sociodemographic index (SDI), a composite indicator derived from the geometric mean of lag-distributed income per capita, average education level for those aged ≥15 years, and the total fertility rate for those aged <25 years^10,15,16^. Based on SDI quintiles, the 204 countries and territories were categorized into five regions: low, low-middle, middle, high-middle, and high SDI. Furthermore, to facilitate age-specific trend analysis, the original 5-year age bin data were aggregated into broader age groups as follows: 40–49 years (combining 40–44 and 45–49), 50–59, 60–69, 70–79, and ≥80 years (comprising 80–84, 85–89, 90–94, and ≥95 years)^11^.

### Statistical analysis

*Descriptive analysis and trend estimation*

Temporal patterns in smoking-attributable LEPAD burden over the period 1990–2021 were evaluated by computing the proportional change in the absolute counts of deaths and DALYs between the baseline and final study years. To further characterize annual trends in age-standardized rates (ASR), we applied the estimated annual percentage change (EAPC) metric. A log-linear regression was fitted with the natural logarithm of the ASR as the dependent variable and calendar year (x) as the independent variable, expressed as: ln (ASR) = α + βx + ϵ, where α denotes the intercept, β the slope coefficient corresponding to the annual rate of change, and ϵ the residual error. From the estimated slope, the EAPC, along with its 95% confidence interval (CI), was obtained using the transformation: EAPC = 100 × [exp(β) – 1]. A statistically significant increasing trend was indicated when both the EAPC point estimate and the lower bound of its 95% CI exceeded zero, whereas a significant decreasing trend was identified when the EAPC and the upper bound of its 95% CI were both below zero. The trends were interpreted based on the EAPC value and its 95% CI: an EAPC with a lower boundary of the 95% CI > 0 indicates a significant increasing trend, whereas an upper boundary <0 indicates a significant decreasing trend.

*Joinpoint regression analysis*

To identify specific time points where the trends in ASRs significantly shifted, this study performed joinpoint regression analysis using the Joinpoint Regression Program (Version 5.1.0.0, Statistical Research and Applications Branch, National Cancer Institute)^17^. This method identifies the optimal number of inflection points (joinpoints) using a grid search method and permutation tests. For each identified segment, this study calculated the annual percentage change (APC) to describe the slope of the trend. To summarize the trend over the entire study period (1990–2021), the average annual percentage change (AAPC) was computed as a weighted average of the APCs from the joinpoint model, with weights equal to the length of each segment.

*Age-period-cohort (APC) analysis*

To examine the independent effects of chronological age, time period, and birth cohort on the disease burden, this study constructed an age-period-cohort (APC) model. The input data were arranged into consecutive 5-year age groups (ranging from 40–44 to ≥80 years) and 5-year time periods (from 1990–1994 through 2015–2019, with 2020–2021 data adjusted to align with model constraints). This analysis focused on estimating several key parameters: Net drift, which represents the overall annual percentage change in the expected age-adjusted rates over time (analogous to the EAPC but adjusted for age and cohort distributions); Local drift, which captures the annual percentage change in rates for each specific age group; and the Longitudinal age curve, which reflects the fitted age-specific rates in the reference cohort adjusted for period effects to represent the biological risk associated with aging. Additionally, the model estimated the period effect (expressed as relative risk, RR), reflecting risk variations over time influenced by immediate factors affecting all age groups simultaneously (e.g. changes in diagnostic criteria or treatments), and the cohort effect (expressed as RR), which highlights risk variations across birth cohorts representing early-life exposures and generational lifestyle differences. Both period and cohort effects were referenced to their respective medians. To address the inherent collinearity problem (age = period − cohort), this study utilized the intrinsic estimator method to ensure parameter identifiability^18^.

*Statistical software and significance*

All statistical analyses were performed using R software (Version 4.3.1, R Foundation for Statistical Computing)^19^. The APC analysis was conducted using the Epi package or comparable tools within the R environment, and p<0.05 was considered statistically significant. All statistical tests were two-sided. The 95% uncertainty intervals (UIs) provided by the GBD study were reported for absolute numbers and rates, while 95% confidence intervals (CIs) were calculated for trend estimates (EAPC, APC, and AAPC).

## RESULTS

### Global burden and temporal trends

In 2021, the global number of deaths due to LEPAD attributable to smoking reached 14130.84 (95% UI: 10300.51–18587.81), and DALYs reached 439653.32 (95% UI: 306683.31–602207.38). Compared to 1990, the absolute number of deaths and DALYs increased by 32.25% (95% UI: 6.74–63.72) and 41.01% (95% UI: 16.0–66.53), respectively (Tables 1 and 2).

**Table 1:** Global trends in mortality of lower extremity peripheral arterial disease attributable to smoking by sex, SDI, and WHO regions from 1990 to 2021

	*Deaths*		*All-age mortality*		*Age-standardized mortality*		*Net drift of mortality per year % (95% CI)*
	*2021 (95% UI)*	*Change 1990 to 2021 (95% UI)*	*Rate in 2021 per 100000 (95% UI)*	*Change 1990 to 2021 % (95% UI)*	*Rate in 2021 per 100000 (95% UI)*	*Change 1990 to 2021 % (95% UI)*	
**Global**	14130.84 (10300.51–18587.81)	32.25 (6.74–63.72)	0.18 (0.13–0.23)	-12.51 (-29.39–8.31)	0.16 (0.12–0.22)	-47.25 (-57.98 – -34.11)	-2.25 (-2.35 – -2.16)
**Sex**							
Male	10986.02 (8105.67–14284.12)	32.56 (7.72–64.3)	0.28 (0.20–0.36)	-12.02 (-28.5–9.04)	0.28 (0.20–0.37)	-49.67 (-59.43 – -37.25)	-2.42 (-2.54 – -2.30)
Female	3144.82 (2187.08–4655.40)	31.19 (-5.16–80.32)	0.08 (0.06–0.12)	-13.49 (-37.46–18.91)	0.07 (0.05–0.10)	-47.96 (-63.55 – -27.8)	-1.97 (-2.17 – -1.77)
**SDI**							
High	10600.59 (7884.78–13891.29)	11.93 (-7.23–35.14)	0.42 (0.31–0.55)	-9.37 (-24.88–9.43)	0.24 (0.18–0.32)	-47.68 (-56.84 – -36.58)	-2.17 (-2.29 – -2.05)
High-middle	1259.69 (885.89–1667.51)	117.97 (58.35–194.08)	0.08 (0.05–0.10)	51.96 (10.40–105.03)	0.07 (0.05–0.09)	-37.37 (-56.35 – -12.04)	-0.33 (-0.7–0.04)
Middle	515.20 (348.30–730.35)	227.27 (117.99–407.08)	0.05 (0.04–0.08)	106.59 (37.60–220.09)	0.07 (0.04–0.09)	11.52 (-25.38–72.86)	-0.10 (-0.74–0.54)
Low-middle	694.30 (408.63–1105.68)	211.74 (62.41–497.25)	0.06 (0.04–0.10)	89.26 (-1.40–262.61)	0.09 (0.05–0.15)	18.04 (-40.54–127.78)	0.63 (0.05–1.20)
Low	1032.81 (555.76–1803.66)	304.54 (87.33–778.96)	0.06 (0.03–0.11)	87.69 (-13.09–307.8)	0.14 (0.07–0.24)	53.12 (-30.22–235.28)	1.43 (0.92–1.94)
**WHO Regions**							
African	962.7 (521.02–1673.95)	186.83 (41.35–451.96)	0.08 (0.04–0.14)	18.94 (-41.39–128.87)	0.20 (0.10–0.35)	6.37 (-49.99–113.68)	0.05 (-0.43–0.53)
Eastern Mediterranean	201.52 (114.10–337.82)	218.6 (47.38–574.62)	0.03 (0.02–0.05)	55.48 (-28.08–229.21)	0.05 (0.03–0.09)	11.66 (-49.72–137.94)	0.53 (-0.44–1.51)
European	6907.99 (5179.93–9055.39)	-4.80 (-23.49–19.24)	0.73 (0.55–0.96)	-13.45 (-30.44–8.41)	0.40 (0.30–0.52)	-41.19 (-52.84 – -26.26)	-2.00 (-2.14 – -1.86)
Americas	3768.78 (2710.09–4996.14)	72.71 (46.00–107.72)	0.37 (0.27–0.49)	20.23 (1.63–44.60)	0.28 (0.20–0.36)	-27.21 (-38.44 – -13.07)	-1.61 (-1.80 – -1.41)
South-East Asia	1104.32 (633.21–1709.12)	326.44 (113.79–789.25)	0.06 (0.04–0.10)	159.84 (30.27–441.85)	0.08 (0.05–0.13)	39.91 (-30.56–195.86)	1.25 (0.74–1.76)
Western Pacific	1134.33 (828.75–1487.60)	88.16 (41.84–143.51)	0.05 (0.04–0.07)	46.09 (10.13–89.07)	0.04 (0.03–0.05)	-41.03 (-55.24 – -23.71)	-1.53 (-1.92 – -1.15)

SDI: sociodemographic index. UI: uncertainty interval. CI: confidence interval. Net drift represents the overall annual percentage change of the age-standardized mortality rate, capturing the sum of linear period and cohort effects. Data source: Global Burden of Disease Study 2021.

**Table 2:** Global trends in DALYs of lower extremity peripheral arterial disease attributable to smoking by sex, SDI, and WHO regions from 1990 to 2021

	*Disability*		*All-age DALYs*		*Age-standardized DALYs*		*Net drift of DALYs per year % (95% CI)*
	*2021 (95% UI)*	*Change 1990 to 2021 (95% UI)*	*Rate in 2021 per 100000 (95% UI)*	*Change 1990 to 2021 % (95% UI)*	*Rate in 2021 per 100000 (95% UI)*	*Change 1990 to 2021 % (95% UI)*	
**Global**	439653.32 (306683.31–602207.38)	41.01 (16.04–66.53)	5.55 (3.87–7.60)	-6.71 (-23.23–10.17)	5.01 (3.48–6.90)	-40.94 (-51.62 – -29.33)	-1.86 (-1.95 – -1.78)
**Sex**							
Male	334336.44 (233469.22–445616.29)	41.32 (19.45–68.79)	8.41 (5.87–11.20)	-6.21 (-20.72–12.03)	8.17 (5.64–10.95)	-42.87 (-52.22 – -31.60)	-2.02 (-2.12 – -1.92)
Female	105316.88 (68730.99–158149.33)	40.05 (6.36–82.40)	2.67 (1.74–4.01)	-7.65 (-29.86–20.28)	2.25 (1.47–3.36)	-40.78 (-54.93 – -22.48)	-1.64 (-1.73 – -1.55)
**SDI**							
High	296085.44 (216059.93–390256.4)	16.64 (-1.62–37.27)	11.70 (8.54–15.42)	-5.55 (-20.34–11.15)	6.94 (5.11–9.08)	-42.78 (-51.84 – -32.62)	-1.89 (-2 – -1.78)
High-middle	55993.16 (36547.37–85824.31)	109.64 (67.38–163.02)	3.46 (2.26–5.30)	46.16 (16.7–83.37)	2.91 (1.88–4.47)	-34.46 (-48.15 – -16.94)	-0.14 (-0.28–0)
Middle	23588.08 (15068.09–35696.22)	168.68 (112.92–258.46)	2.49 (1.59–3.77)	69.60 (34.41–126.28)	2.95 (1.85–4.52)	-6.69 (-26.94–23.46)	-0.47 (-0.58 – -0.37)
Low-middle	28272.08 (16607.89–43160.67)	138.34 (60.31–260.46)	2.43 (1.43–3.72)	44.70 (-2.67–118.84)	3.42 (1.99–5.33)	-7.93 (-38.14–40.39)	0.11 (-0.01–0.23)
Low	34999.37 (20117.68–57021.37)	225.25 (96.36–459.82)	2.11 (1.21–3.43)	50.9 (-8.90–159.73)	4.32 (2.46–6.99)	25.08 (-22.45–113.47)	0.93 (0.81–1.06)
**WHO Regions**							
African	27094.05 (15163.23–43932.07)	178.94 (45.03–412.43)	2.31 (1.30–3.75)	15.67 (-39.86–112.48)	5.06 (2.81–8.27)	6.06 (-45.88–96.17)	-0.16 (-0.37–0.05)
Eastern Mediterranean	10726.69 (6083.13–17946.13)	163.27 (68.90–305.11)	1.44 (0.82–2.41)	28.48 (-17.58–97.69)	2.61 (1.41–4.41)	-7.72 (-41.42–41.01)	0.09 (-0.06–0.25)
European	177265.38 (128755.09–232003.53)	-3.81 (-21.78–17.5)	18.76 (13.62–24.55)	-12.55 (-28.89–6.83)	10.81 (7.97–14.01)	-37.99 (-49.26 – -23.94)	-1.89 (-2.02 – -1.75)
Americas	101294.13 (71791.68–134302.4)	67.25 (43.05–95.46)	9.93 (7.04–13.16)	16.43 (-0.42–36.07)	7.46 (5.31–9.80)	-28.11 (-38.35 – -15.99)	-1.51 (-1.58 – -1.44)
South-East Asia	46385.77 (26253.86–76073.94)	179.61 (95.2–314.68)	2.59 (1.47–4.25)	70.37 (18.94–152.68)	3.24 (1.83–5.31)	-7.80 (-36.33–40.01)	0.27 (0.11–0.42)
Western Pacific	75208.44 (41042.45–128517.79)	104.13 (68.23–139.06)	3.40 (1.86–5.81)	58.49 (30.62–85.61)	2.37 (1.30–4.03)	-30.50 (-42.54 – -18.13)	-0.91 (-1.03 – -0.80)

DALYs: disability-adjusted life years. SDI: sociodemographic index. UI: uncertainty interval. CI: confidence interval. The all-age DALYs rate is equivalent to the crude DALYs rate. Net drift represents the estimated overall annual percentage change in DALYs, adjusting for period and cohort effects. Data source: Global Burden of Disease Study 2021.

Despite the increase in absolute numbers, the age-standardized rates exhibited a consistent downward trend over the past three decades. From 1990 to 2021, the global ASMR decreased by 47.25% (95% UI: 34.11–57.98) to 0.16 (95% UI: 0.12–0.22) per 100000 population, and the ASDR decreased by 40.94% (95% UI: 29.33–51.62) to 5.01 (95% UI: 3.48–6.90) per 100000 population. The estimated Net drift for mortality and DALYs was -2.25% (95% CI: -2.35 – -2.16) and -1.86 (95% CI: -1.95 – -1.78) per year, respectively, indicating a significant overall improvement in the disease burden after adjusting for age and cohort effects.

Substantial gender disparities were observed in the disease burden. In 2021, the absolute number of deaths in males (10986.02) was approximately 3.5 times higher than that in females (3144.82), and DALYs in males (334336.44) were more than three times higher than in females (105316.88). Similarly, the ASMR and ASDR for males (0.28 and 8.17 per 100000, respectively) were markedly higher than those for females (0.07 and 2.25 per 100000, respectively). However, both sexes showed significant declining trends in age-standardized rates from 1990 to 2021, with males experiencing a slightly larger percentage decrease in ASMR (-49.67%) compared to females (-47.96%) (Tables 1 and 2).

### Burden of LEPAD attributable to smoking across different SDI regions

**Figure 1:**
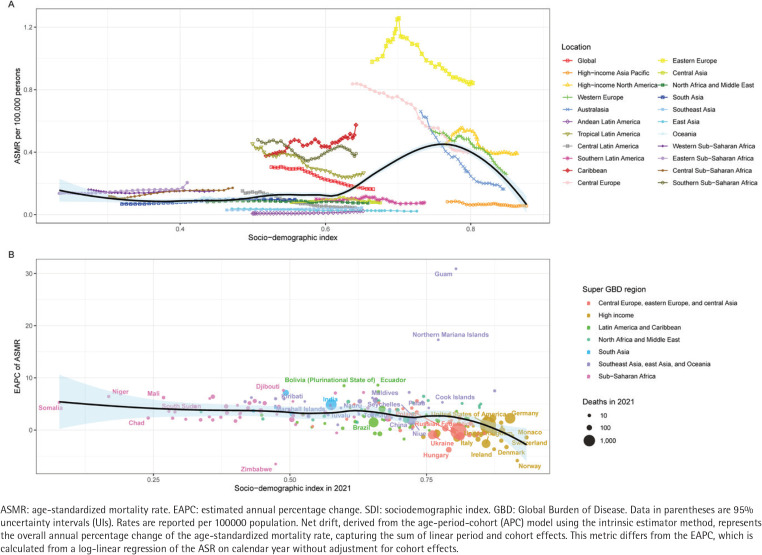
The association between the burden of lower extremity peripheral arterial disease attributable to smoking and SDI from 1990 to 2021: A) The trends in ASMR across 21 GBD regions. Colored lines represent the trajectories of different regions over time. The black solid line represents the expected ASMR based on SDI values across all regions; B) The correlation between the EAPC of ASMR and the SDI in 2021 across 204 countries and territories. Circles represent countries or territories, with the size proportional to the absolute number of deaths in 2021. The colors of the circles denote the seven GBD super-regions. The black line represents the polynomial regression fitting curve, and the blue shaded area indicates the 95% confidence interval

This study examined the relationship between the age-standardized burden (ASMR and ASDR) of LEPAD attributable to smoking and the SDI across 21 GBD regions from 1990 to 2021 (Figure 1A; and Supplementary file Figure 1A). Generally, the expected values of ASMR and ASDR (represented by the black solid line) exhibited a non-linear relationship with SDI. The burden remained relatively stable or increased slightly in regions with low to middle SDI, but showed a sharp decline as SDI values exceeded approximately 0.7. However, substantial regional heterogeneity was observed. Notably, Eastern Europe exhibited observed ASMR and ASDR values that were significantly higher than the expected levels based on their SDI throughout the study period. In contrast, regions such as high-income Asia Pacific and Australasia generally followed the expected declining trajectory associated with high SDI improvement.

A significant negative correlation was observed between the EAPC of the disease burden and the SDI in 2021 at the national level (Figure 1B; and Supplementary file Figure 1B). The polynomial regression fitting curves (black lines) for both mortality and DALYs demonstrated that countries with low SDI values (typically <0.7) predominantly experienced an increasing trend in disease burden (EAPC >0). Conversely, countries with high SDI values (typically >0.75) mostly exhibited a decreasing trend (EAPC <0). Specifically, in high-SDI regions (represented by yellow and orange circles), such as those in Western Europe and high-income North America, the EAPC values were largely negative, indicating effective control of the smoking-attributable LEPAD burden. In contrast, many countries in Sub-Saharan Africa and South Asia (represented by pink and blue circles) showed positive EAPCs, suggesting a rising burden in these developing regions.

### The independent effects of age, period, and cohort on the burden of LEPAD attributable to smoking

**Figure 2:**
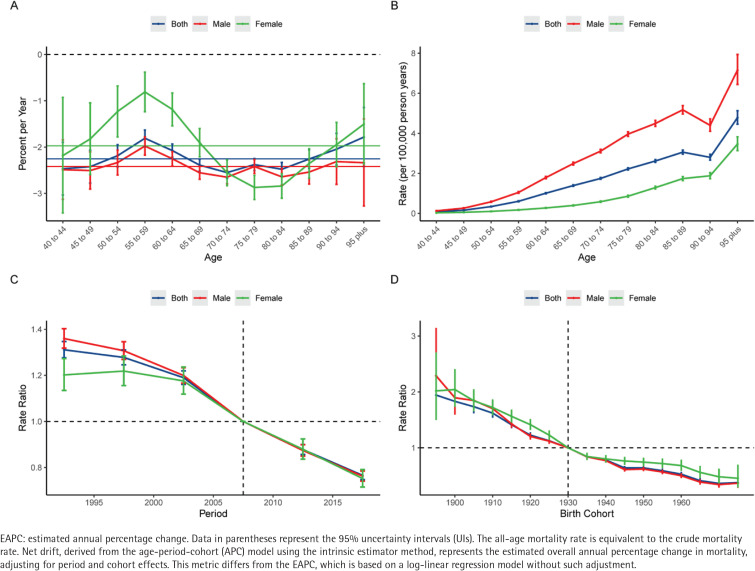
Age-period-cohort analysis of the global burden of lower extremity peripheral arterial disease mortality attributable to smoking from 1990 to 2021: A) Local drift – the annual percentage change of the rates for each age group (5-year intervals). The horizontal solid lines represent the net drift (overall estimated annual percentage change) for the corresponding sex; B) Age effects – the longitudinal age curves of the mortality rates (per 100000 population), adjusted for period and cohort effects; C) Period effects – the relative risk (rate ratio) of mortality over time, adjusted for age and cohort effects; D) Cohort effects – the relative risk (rate ratio) of mortality across different birth cohorts, adjusted for age and period effects. Blue, red, and green lines represent both sexes, males, and females, respectively. Shaded areas and error bars denote 95% confidence interval. The dashed horizontal line indicates a rate ratio of 1.0

**Figure 3:**
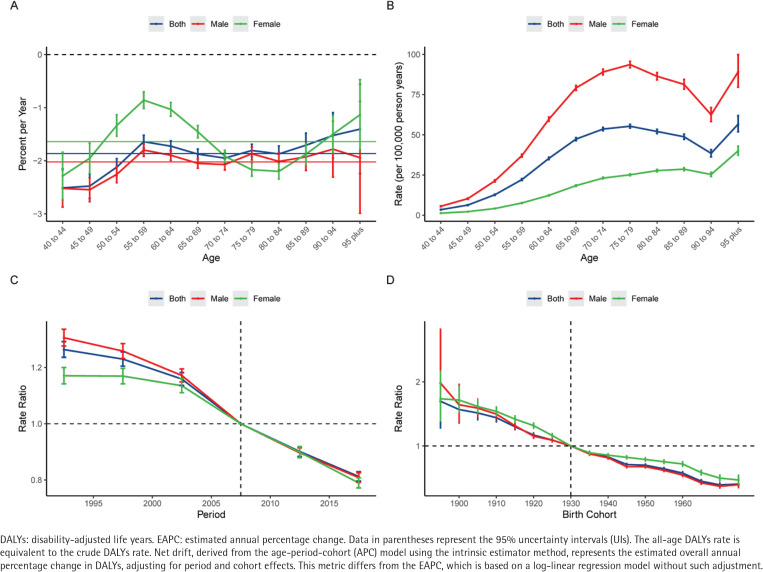
Age-period-cohort analysis of the global burden of lower extremity peripheral arterial disease DALYs attributable to smoking from 1990 to 2021: A) Local drift – the annual percentage change of the rates for each age group (5-year intervals). The horizontal solid lines represent the net drift (overall estimated annual percentage change) for the corresponding sex; B) Age effects – the longitudinal age curves of the DALY rates (per 100000 population), adjusted for period and cohort effects; C) Period effects – the relative risk (rate ratio) of DALYs over time, adjusted for age and cohort effects; D) Cohort effects – the relative risk (rate ratio) of DALYs across different birth cohorts, adjusted for age and period effects. Blue, red, and green lines represent both sexes, males, and females, respectively. Shaded areas and error bars denote 95% confidence interval. The dashed horizontal line indicates a rate ratio of 1.0

The age-period-cohort model was utilized to delineate the independent effects of age, period, and birth cohort on the mortality and DALYs of LEPAD attributable to smoking. The estimated local drift values for both mortality and DALYs were consistently below zero across all age groups (aged 40–95 plus) for both sexes, indicating a universal amelioration in the disease burden over the study period (Figures 2A and 3A). For mortality, the most significant annual percentage decline for males was observed in the age group of 70–74 years (-2.65%; 95% CI: -2.79 – -2.51), whereas for females, the nadir occurred in the age group of 75–79 years (-2.87%; 95% CI: -3.13 – -2.61). Regarding DALYs, the most rapid decline for males was in the 45–49 years age group (-2.55%; 95% CI: -2.77 – -2.32), while for females, the improvement was most pronounced in the age group of 80–84 years (-2.20%; 95% CI: -2.34 – -2.05). Notably, in the middle-aged groups (50–60 years), the pace of decline in DALYs slowed down, particularly among females.

The longitudinal age curves demonstrated that the risk of LEPAD mortality and DALYs attributable to smoking increased exponentially with age (Figures 2B and 3B). Generally, males bear a substantially heavier burden than females across all age groups. For mortality, the rates for males rose sharply from 0.13 per 100000 at age 40–44 years to a peak of 7.15 per 100000 in the ≥95 years age group, significantly higher than the female peak of 4.78 per 100000 in the same age group. Interestingly, the age pattern for DALYs differed slightly; male DALYs rates peaked in the age group of 75–79 years (93.64 per 100000) before declining in the oldest ages, whereas female DALYs rates showed a continuous upward trend, reaching 40.06 per 100000 in the ≥95 years age group.

Regarding the period effects, the relative risk (RR) of mortality and DALYs for both sexes exhibited a monotonic downward trend from 1990 to 2021 (Figures 2C and 3C). The RR of mortality for the total population decreased from 1.31 (95% CI: 1.28–1.35) in the period 1990–1994 to 0.77 (95% CI: 0.75–0.78) in 2015–2019. Similarly, the RR of DALYs declined from 1.26 (95% CI: 1.24–1.29) to 0.81 (95% CI: 0.80–0.83) over the same intervals. This consistent decline suggests that advancements in medical management and tobacco control policies have effectively mitigated the risk over the past three decades.

The cohort effects revealed a progressive decrease in the risk of death and disability for successive birth cohorts (Figures 2D and 3D). Individuals born in earlier cohorts faced a much higher risk compared to those born in later cohorts. For instance, the RR of mortality for the 1895 birth cohort was 1.94 (95% CI: 1.57–2.40), which dropped substantially to 0.38 (95% CI: 0.31–0.45) for the 1975 birth cohort. A similar descending pattern was observed for DALYs, with the RR decreasing from 1.70 (95% CI: 1.28–2.25) in the 1895 cohort to 0.41 (95% CI: 0.36–0.46) in the 1975 cohort. This trend was consistent for both males and females, although males in the earliest birth cohorts included in the APC analysis (i.e. those born before 1910, spanning approximately the 1895–1910 birth cohorts) initially exhibited a higher relative risk than females.

### Joinpoint regression analysis of global trends in the burden of LEPAD attributable to smoking

The joinpoint regression analysis was employed to examine the temporal trends in the age-standardized rates of LEPAD attributable to smoking from 1990 to 2021. Generally, both the ASMR and ASDR exhibited a significant downward trend over the past three decades (Supplementary file Figure 2).

For the global population, the ASMR declined with an average annual percentage change (AAPC) of -1.98% (Supplementary file Figure 2A). The trend can be divided into four distinct phases, with the most rapid decline occurring between 2000 and 2007, characterized by an annual percentage change (APC) of -3.43% (p<0.05). Similarly, the global ASDR decreased with an AAPC of -1.60%, also showing the most significant reduction during the 2000–2007 period (APC= -2.85%, p<0.05) (Supplementary file Figure 2B).

In terms of gender, males generally exhibited a slightly faster overall decline in mortality than females. The ASMR for males decreased with an AAPC of -2.14% (Supplementary file Figure 2C), compared to -1.99% for females (Supplementary file Figure 2D). For males, the mortality trend mirrored the global pattern, with the steepest decline observed from 2000 to 2007 (APC= -3.62%, p<0.05). In contrast, the trend for females was more complex; the ASMR remained relatively stable from 1990 to 2000 (APC=0.07%, p>0.05), followed by a precipitous drop, particularly between 2003 and 2006, where the APC reached -4.62% (p<0.05).

Regarding the burden of disability, the AAPC for ASDR was -1.72% for males and -1.57% for females (Supplementary file Figures 2E and 2F). The decline in male ASDR was most pronounced between 2000 and 2008 (APC= -3.01%, p<0.05). For females, the trend was characterized by two main phases: a slow and insignificant decline from 1990 to 2000 (APC= -0.11%), followed by a consistent and significant downward trend from 2000 to 2021 (APC= -2.25%, p<0.05). Collectively, these findings indicate that while the burden has decreased for both sexes, the acceleration of this decline began largely after the year 2000.

### Age-specific and sex-specific burden of LEPAD attributable to smoking

The distribution of the disease burden shifted noticeably toward older age groups from 1990 to 2021, reflecting global population aging, even though age-specific rates for both mortality and DALYs consistently declined across almost all age categories (Supplementary file Figure 3). Regarding sex disparities, males consistently exhibited significantly higher mortality and DALY rates than females across all age groups, with the female burden being notably more concentrated in the oldest age category (aged ≥80 years) (Supplementary file Figures 4 and 5).

### National level burden of LEPAD attributable to smoking

The global spatial distribution of the LEPAD burden attributable to smoking in 1990 and 2021 is illustrated in Supplementary file Figure 6, revealing substantial geographical heterogeneity. In 2021, regions with the highest age-standardized rates were predominantly concentrated in Eastern Europe, the Caribbean, and parts of Central Asia, indicated by the red areas in the maps (Supplementary file Figures 6B and 6D). Supplementary file Table 1 provides specific estimates confirming these patterns. In 2021, Cuba recorded the highest age-standardized mortality rate (ASMR) at 1.20 (95% UI: 0.80–1.70) per 100000 population, followed by Belarus (1.10; 95% UI: 0.80–1.40) and the Russian Federation (0.90; 95% UI: 0.70–1.30). Regarding morbidity, Belarus exhibited the highest age-standardized DALY rate (ASDR) at 27.9 (95% UI: 21.50–36.20) per 100000, followed by Cuba (27.20; 95% UI: 18.70–37.00) and Micronesia (25.60; 95% UI: 15.40–40.30). Conversely, the burden remained lowest in Sub-Saharan Africa and Andean Latin America (blue areas in Supplementary file Figure 6), with countries such as Ethiopia and Peru reporting ASMRs and ASDRs near zero. Notably, a significant reduction in burden was observed in several high-income nations over the study period. For instance, the ASMR in Ireland plummeted from 1.20 (95% UI: 0.90–1.60) in 1990 to 0.20 (95% UI: 0.20–0.30) in 2021, and in the United Kingdom, it decreased from 1.00 (95% UI: 0.70–1.30) to 0.30 (0.20–0.50), reflecting the shifting epidemiological landscape depicted in the temporal comparison of the maps.

## DISCUSSION

This study provides the most up-to-date and comprehensive assessment of the spatiotemporal trends in the global burden of LEPAD attributable to smoking over the past three decades based on the GBD 2021 study. Our findings reveal a divergent trend: while the absolute number of deaths and DALYs has increased substantially – driven largely by global population growth and aging – the ASMR and ASDR exhibited a remarkable decline of approximately 47% and 41%, respectively, from 1990 to 2021. By utilizing the age-period-cohort model, this study further disentangled these trends and identified significant favorable changes in both period and cohort effects. Specifically, the relative risk of LEPAD burden has consistently decreased across successive birth cohorts and calendar periods, reflecting the cumulative benefits of global tobacco control and medical advancements. However, despite these encouraging overall reductions, the burden remains unequally distributed. This study observed pronounced gender disparities, with males bearing a significantly heavier burden than females, alongside substantial heterogeneity across different SDI regions and countries, highlighting the persistent challenge of health inequality in addressing smoking-related LEPAD.

The substantial decline in age-standardized mortality and DALY rates over the past three decades is likely driven by the synergistic effect of global tobacco control initiatives and advancements in cardiovascular medicine. Our joinpoint analysis identified a pivotal acceleration in the downward trend of disease burden after the year 2000, particularly distinguishing the 2000–2007 period. This timeline coincides remarkably with the adoption of the WHO Framework Convention on Tobacco Control (FCTC) in 2003 and the subsequent global rollout of MPOWER strategies^20,21^. The rigorous enforcement of tobacco control measures – such as increased taxation, comprehensive smoke-free legislation, and graphic health warnings on packaging – has been proven to effectively reduce smoking prevalence and intensity, thereby directly lowering the incidence of smoking-related vascular diseases^22^. Concurrently, improvements in the clinical management of LEPAD and its comorbidities have significantly contributed to reduced lethality and disability among smokers. The widespread prescription of lipid-lowering agents (statins), antiplatelet therapy, and optimal control of hypertension and diabetes has improved the overall prognosis of atherosclerotic diseases. Moreover, the evolution and accessibility of revascularization techniques, including endovascular interventions, have likely lowered amputation rates and improved the quality of life for symptomatic patients^23^. These macro-environmental improvements are corroborated by the period effects observed in our APC analysis, where the continuous decline in relative risk suggests that advancements in policy and medical care have universally benefited populations across all age groups over the study period.

Despite favorable downward trends in age-standardized rates, the absolute burden of LEPAD attributable to smoking – measured in total deaths and DALYs – has continued to rise significantly over the study period. This apparent paradox is primarily driven by profound global demographic shifts, specifically population growth and rapid aging. As demonstrated by our longitudinal age curves, LEPAD is intrinsically an age-dependent disease, with the risk of mortality and morbidity escalating exponentially with advancing age. Consequently, the expansion of the global geriatric population effectively offsets the gains achieved through reduced smoking prevalence and improved medical interventions. This ‘aging effect’ poses an important challenge to public health systems worldwide. Even if tobacco control strategies successfully lower the incidence rate, the sheer volume of older patients requiring complex vascular care, is projected to increase. The strain is expected to be particularly acute in low- and middle-income countries (LMICs), where the pace of population aging is accelerating faster than in high-income nations, yet healthcare infrastructure for managing chronic vascular conditions often remains inadequate^24^.

A striking gender disparity characterizes the burden of smoking-attributable LEPAD, with males bearing approximately 3.5 times the mortality and DALYs burden of females in 2021. This inequality is primarily attributable to the historical gender gap in tobacco consumption, where men have consistently exhibited higher smoking prevalence and greater cumulative exposure (pack-years) over the past century^5^. Biologically, the harmful constituents of tobacco smoke – such as nicotine, carbon monoxide, and oxidizing chemicals – accelerate atherothrombosis by inducing endothelial dysfunction, systemic inflammation, and platelet activation^7^. While higher exposure explains the bulk of the male burden, emerging evidence suggests a potential ‘female disadvantage’ hypothesis, indicating that women might be biologically more susceptible to the vascular toxicity of tobacco per unit of exposure compared to men^25^. Furthermore, our trend analysis revealed that the decline in ASMR was slightly more pronounced in males than in females. This discrepancy warrants caution regarding the distinct stages of the tobacco epidemic. In some developing regions and low-to-middle SDI countries, female smoking rates are stabilizing or even rising due to shifting sociocultural norms and targeted marketing, a phenomenon known as the ‘catch-up’ effect^26^. If this trend persists, the gender gap in LEPAD burden may narrow in a detrimental way, necessitating gender-specific tobacco control strategies.

The independent cohort effects derived from our APC model reveal a continuous and steep decline in LEPAD risk across successive birth cohorts, with the 1975 cohort exhibiting a substantially lower relative risk compared to the 1895 cohort. This intergenerational improvement is likely a reflection of fundamental shifts in socio-economic and educational environments. Newer generations have generally benefited from higher levels of education and health literacy, factors that are strongly inversely associated with smoking initiation and maintenance^27^. Unlike earlier cohorts who reached maturity when smoking was socially widespread and normalized, recent cohorts were raised during an era of intensifying anti-tobacco campaigns and widespread public health education. Consequently, they were less likely to initiate smoking at a young age or to sustain heavy consumption^28^. This finding underscores the critical importance of early-life exposure in the pathogenesis of LEPAD. Since peripheral atherosclerosis is a chronic, cumulative process that often begins in youth, preventing smoking uptake during adolescence translates directly into a reduced lifetime accumulation of vascular damage and oxidative stress^29^. Thus, the diminishing risk in later cohorts provides robust epidemiological evidence validating the long-term effectiveness of youth-focused tobacco prevention strategies.

Substantial geographical heterogeneity exists in the burden of smoking-attributable LEPAD, following a distinct sociodemographic gradient. Our analysis reveals a significant negative correlation between the estimated annual percentage change (EAPC) and the SDI. High-SDI regions, such as Western Europe and high-income North America, have achieved marked reductions in disease burden, reflecting the success of mature public health infrastructures and tobacco control policies. In stark contrast, the burden in Eastern Europe and Central Asia remains disproportionately high, with countries like Belarus and the Russian Federation recording some of the highest age-standardized rates globally. This regional ‘hotspot’ is likely driven by the historically profound prevalence of smoking among men, exacerbated by heavy alcohol consumption^30^. Epidemiological evidence suggests a synergistic detriment between smoking and alcohol on vascular health, where their combined effect on raising blood pressure and inducing endothelial injury exceeds the sum of their individual risks^31^. Conversely, Sub-Saharan Africa currently presents a lower burden, largely due to its younger demographic structure. However, the positive EAPC observed in several nations in this region signals an emerging epidemic. This rising trend is alarming and likely reflects the aggressive expansion strategies of transnational tobacco companies targeting developing markets to replace declining sales in high-income countries^32^. Without pre-emptive intervention, these regions risk facing a surge in LEPAD cases as their populations age and cumulative exposure increases.

The findings of this study may have important implications for global public health policy and clinical practice. First, precision prevention strategies may be warranted to address the uneven distribution of disease burden. Given the exceptionally high mortality and DALY rates observed in Eastern Europe and Central Asia, as well as the concentrated risk among older males, future efforts could consider targeted screening programs, such as those using the Ankle-Brachial Index (ABI) in these high-prevalence populations. Early detection of asymptomatic LEPAD in high-risk groups is a cost-effective measure to prevent critical limb ischemia and amputation^33^. Second, despite the progress made, smoking remains the single most modifiable risk factor for LEPAD. Sustained and accelerated implementation of the WHO FCTC strategies may play an important role, particularly in low- and middle-income countries where tobacco taxation and marketing bans are often under-enforced^34^. Finally, for individuals with a history of smoking, cessation alone may not fully mitigate the risk of advanced atherosclerosis due to the ‘legacy effect’ of prior exposure. Therefore, a comprehensive management approach that integrates smoking cessation with aggressive control of hypertension and dyslipidemia – specifically through the use of statins and antiplatelet therapy – could be beneficial to minimize the residual cardiovascular risk in this vulnerable cohort^35^.

### Limitations

Our study is subject to several limitations inherent to the GBD methodology. First, the accuracy of our estimates relies heavily on the availability and quality of primary data sources. For regions with sparse vital registration systems or limited epidemiological surveys – such as parts of Sub-Saharan Africa and Oceania – the GBD framework utilizes predictive modeling (e.g. DisMod-MR 2.1) to generate estimates based on covariates and regional patterns. While robust, these mathematical extrapolations may not fully capture the granular variations in local disease burden. Second, the exposure data for smoking are primarily derived from self-reported surveys, which are susceptible to recall bias and social desirability bias, potentially leading to an underestimation of the true prevalence. Furthermore, the current analysis focused predominantly on combustible tobacco products. The emerging vascular impact of other nicotine products, such as electronic cigarettes and heated tobacco products, remains under-characterized in long-term historical data and warrants urgent investigation in future GBD cycles. Third, as an aggregate-level ecological analysis, our findings describe population-level associations and cannot directly infer individual causality between smoking patterns and LEPAD outcomes (ecological fallacy). Finally, despite the application of the Meta-Regression-Bayesian, Regularized, Trimmed (MR-BRT) tool to adjust non-reference data (e.g. administrative claims using ICD codes) to the reference standard (Ankle-Brachial Index ≤0.9), residual heterogeneity in diagnostic criteria across different healthcare systems may still influence the comparability of LEPAD prevalence. Future research should prioritize the establishment of standardized peripheral vascular registries and the incorporation of biomarkers to refine burden estimation.

## CONCLUSIONS

This study demonstrates a significant decline in the age-standardized burden of LEPAD attributable to smoking over the past three decades, driven by global tobacco control initiatives and advancements in vascular medicine. However, the absolute burden continues to rise due to global population aging, accompanied by persistent disparities across genders and regions, particularly in Eastern Europe, Central Asia, and among older males. Consequently, targeted intervention strategies tailored to specific high-risk regions and populations may be warranted. Furthermore, integrating robust tobacco control measures with comprehensive chronic cardiovascular disease management may be important and could contribute to addressing this challenge.

## Supplementary Material



## Data Availability

The data supporting this research are available from the authors on reasonable request.
