# Supplementary material for: Global burden of lower extremity peripheral arterial disease attributable to smoking from 1990 to 2021: A secondary dataset analysis of the Global Burden of Disease Study 2021
Source: Tob Induc Dis. 2026 May 7;24:10.18332/tid/219768. doi: 10.18332/tid/219768 (PMC13154163; doi:10.18332/tid/219768)
Supplement: Supplementary file 1 [file TID-24-55-s1.pdf]

Global burden of lower extremity peripheral arterial disease attributable to smoking from 1990 to 2021: An age-period-cohort analysis of the Global Burden of Disease Study 2021

SUPPLEMENTARY DATA

Table of Contents

1. Supplementary Table 1. The age-standardized mortality rate (ASMR) and age-standardized disability-adjusted life years rate (ASDR) of lower extremity peripheral arterial disease attributable to smoking in 204 countries and territories in 1990 and 2021. 2
2. Supplementary Figure 1. The association between the disability-adjusted life years (DALYs) burden of lower extremity peripheral arterial disease attributable to smoking and socio-demographic index (SDI) from 1990 to 2021. 8
3. Supplementary Figure 2. Joinpoint regression analysis of global trends in the burden of lower extremity peripheral arterial disease attributable to smoking from 1990 to 2021. 9
4. Supplementary Figure 3. The age-specific proportion and rate changes in the burden of lower extremity peripheral arterial disease attributable to smoking from 1990 to 2021. 10
5. Supplementary Figure 4. The age-specific proportion and rate changes in mortality of lower extremity peripheral arterial disease attributable to smoking by sex from 1990 to 2021. 11
6. Supplementary Figure 5. The age-specific proportion and rate changes in DALYs of lower extremity peripheral arterial disease attributable to smoking by sex from 1990 to 2021 12
7. Supplementary Figure 6. The global spatial distribution of the burden of lower extremity peripheral arterial disease attributable to smoking in 1990 and 2021. 13

1. **Supplementary Table 1.** The age-standardized mortality rate (ASMR) and age-standardized disability-adjusted life years rate (ASDR) of lower extremity peripheral arterial disease attributable to smoking in 204 countries and territories in 1990 and 2021.

	Mortality		DALYs	
	ASMR (per 100,000) in 1990	ASMR (per 100,000) in 2021	ASDR (per 100,000) in 1990	ASDR (per 100,000) in 2021
Afghanistan	0 (0-0)	0 (0-0)	0.5 (0.2-0.9)	0.9 (0.4-1.7)
Albania	0.1 (0-0.1)	0.1 (0-0.1)	3.3 (1.8-5.5)	3.4 (2.1-5.6)
Algeria	0.1 (0-0.1)	0.1 (0-0.1)	3 (1.7-5)	2.3 (1.3-4.1)
American Samoa	0.3 (0.2-0.5)	0.2 (0.1-0.4)	9.4 (5.5-14.6)	7.8 (4.2-13.5)
Andorra	0.4 (0.2-0.6)	0.3 (0.1-0.5)	9.5 (5.7-14.9)	7.8 (4.5-12.4)
Angola	0.2 (0.1-0.4)	0.3 (0.1-0.5)	6.5 (3.1-11)	7.5 (3.8-12.9)
Antigua and Barbuda	0.2 (0.1-0.3)	0.2 (0.1-0.3)	5 (2.8-7.7)	5.3 (3.2-8.3)
Argentina	0.1 (0.1-0.1)	0.1 (0.1-0.1)	3.2 (2-4.7)	2.7 (1.7-4.2)
Armenia	0.6 (0.4-0.8)	0.3 (0.2-0.4)	15.5 (11-20.1)	7.5 (5.6-9.8)
Australia	0.7 (0.5-0.9)	0.2 (0.1-0.2)	13.5 (9.8-17.6)	3.7 (2.4-5.2)
Austria	0.7 (0.5-0.9)	0.4 (0.3-0.5)	15.5 (10.7-20.3)	9.4 (6.7-12.9)
Azerbaijan	0.1 (0-0.1)	0.1 (0-0.1)	2.1 (1.3-3.2)	2.7 (1.6-4.4)
Bahamas	0.2 (0.1-0.3)	0.2 (0.1-0.4)	5.1 (2.9-7.7)	6.6 (3.8-10.8)
Bahrain	0.3 (0.2-0.5)	0.3 (0.2-0.5)	9.1 (5.8-12.9)	7.5 (4.5-11.4)
Bangladesh	0.1 (0-0.1)	0.2 (0.1-0.3)	3.5 (1.7-6.1)	5.4 (2.8-8.6)
Barbados	0.4 (0.2-0.6)	0.3 (0.2-0.6)	10.3 (5.8-15.6)	8.6 (4.7-14.6)
Belarus	1.2 (0.8-1.6)	1.1 (0.8-1.4)	30 (20.8-40.4)	27.9 (21.5-36.2)
Belgium	0.3 (0.2-0.4)	0.1 (0.1-0.2)	8.7 (5.9-12.2)	4.7 (2.8-7.5)
Belize	0.1 (0-0.1)	0.1 (0.1-0.1)	3 (1.7-4.5)	3 (1.7-4.9)
Benin	0.2 (0.1-0.3)	0.1 (0.1-0.3)	4.1 (1.8-7.2)	3.6 (1.6-7)
Bermuda	0.4 (0.2-0.7)	0.3 (0.2-0.6)	10.7 (6.2-16.1)	7.4 (4.6-12.2)
Bhutan	0 (0-0.1)	0 (0-0.1)	2.1 (0.9-3.7)	1.9 (0.8-3.6)
Bolivia (Plurinational State of)	0 (0-0)	0 (0-0.1)	1.1 (0.5-2.1)	1.5 (0.8-2.7)
Bosnia and Herzegovina	0.1 (0-0.2)	0.1 (0.1-0.2)	3.4 (1.8-5.6)	4.4 (2.6-7)
Botswana	0.2 (0.1-0.4)	0.4 (0.2-0.8)	6.7 (3.5-11.3)	11.4 (6.4-18.3)
Brazil	0.5 (0.3-0.6)	0.3 (0.2-0.4)	12.7 (8.8-16.5)	7.5 (5.2-10.1)
Brunei Darussalam	0.1 (0.1-0.2)	0.1 (0-0.1)	4.3 (2.4-6.8)	2.3 (1.3-3.7)
Bulgaria	0.1 (0.1-0.2)	0.1 (0.1-0.1)	3.9 (2.5-5.6)	3.9 (2.8-5.7)
Burkina Faso	0.1 (0-0.2)	0.1 (0-0.2)	2.6 (1.2-4.7)	2.3 (1.1-4.5)
Burundi	0.1 (0-0.2)	0.1 (0.1-0.3)	2.7 (1.3-4.6)	3.4 (1.5-7.1)
Cabo Verde	0.1 (0.1-0.3)	0.2 (0.1-0.3)	3.4 (1.7-5.9)	3.9 (1.9-6.9)
Cambodia	0 (0-0)	0 (0-0.1)	2.5 (1.2-4.3)	2.6 (1.4-4.4)
Cameroon	0.2 (0.1-0.5)	0.2 (0.1-0.5)	5.7 (2.5-10.9)	5.7 (2.8-11)
Canada	0.6 (0.5-0.8)	0.3 (0.2-0.4)	14.2 (9.9-18.9)	7.5 (5.1-10.7)
Central African Republic	0.1 (0-0.3)	0.1 (0-0.3)	3.5 (1.5-6.5)	3.4 (1.4-6.7)

Chad	0.2 (0.1-0.5)	0.2 (0.1-0.5)	5.4 (2.2-10)	5.4 (2.5-10.5)
Chile	0.2 (0.1-0.2)	0.1 (0.1-0.1)	4.3 (2.8-5.9)	2.9 (1.9-4.3)
China	0 (0-0.1)	0 (0-0)	3.4 (1.7-6.4)	2.3 (1.2-4.3)
Colombia	0.1 (0.1-0.2)	0 (0-0.1)	4.4 (2.6-6.6)	1.6 (0.9-2.7)
Comoros	0.1 (0-0.2)	0.1 (0-0.3)	2.7 (1.2-4.6)	2.9 (1.3-6.6)
Congo	0.1 (0-0.2)	0.3 (0.2-0.6)	3.1 (1.5-5.1)	8.4 (3.9-14.8)
Cook Islands	0.1 (0.1-0.2)	0.3 (0.1-0.5)	4.1 (2.3-6.4)	8.8 (4.1-15.7)
Costa Rica	0.1 (0.1-0.2)	0 (0-0.1)	4 (2.3-6.3)	1.8 (0.9-3)
Coted'Ivoire	0.1 (0.1-0.3)	0.2 (0.1-0.4)	3.5 (1.7-6.4)	4.9 (2.3-8.8)
Croatia	0.9 (0.6-1.2)	0.6 (0.4-0.8)	19.8 (14.2-26.3)	14.2 (10.7-19)
Cuba	0.6 (0.4-0.9)	1.2 (0.8-1.7)	14.6 (9.6-20)	27.2 (18.7-37)
Cyprus	0.4 (0.2-0.6)	0.2 (0.1-0.3)	10.1 (6.1-16.2)	5.9 (3.5-9.2)
Czechia	0.6 (0.4-0.8)	0.3 (0.2-0.4)	15.7 (10.8-20.4)	8.7 (6.3-11.6)
Democratic People's Republic of Korea	0 (0-0)	0 (0-0)	1.3 (0.6-2.4)	1.3 (0.7-2.4)
Democratic Republic of the Congo	0.1 (0-0.2)	0.1 (0-0.2)	2.4 (1.1-4.7)	3.3 (1.4-6.3)
Denmark	1.1 (0.8-1.4)	0.3 (0.2-0.4)	23.2 (17.5-29.3)	7.7 (5.3-10.9)
Djibouti	0.2 (0.1-0.4)	0.3 (0.1-0.8)	4.9 (2.2-9.1)	8 (3.9-17.8)
Dominica	0.1 (0.1-0.2)	0.2 (0.1-0.3)	3.5 (1.8-5.5)	5.1 (2.9-8.4)
Dominican Republic	0.2 (0.1-0.3)	0.1 (0.1-0.2)	5.8 (3.1-9.1)	3.6 (2-5.7)
Ecuador	0 (0-0)	0 (0-0)	1.5 (0.7-2.8)	1.1 (0.6-2)
Egypt	0 (0-0)	0 (0-0.1)	1.6 (0.8-2.7)	2.2 (1.2-3.8)
El Salvador	0 (0-0.1)	0 (0-0.1)	1.7 (0.8-3)	1.1 (0.5-1.9)
Equatorial Guinea	0.1 (0.1-0.3)	0.3 (0.2-0.6)	4 (1.8-7.4)	8.6 (4.4-16.6)
Eritrea	0 (0-0.1)	0.1 (0-0.1)	1.2 (0.5-2.5)	1.6 (0.7-3.8)
Estonia	0.4 (0.3-0.6)	0.3 (0.2-0.4)	12.1 (8.6-16)	7.5 (5.4-9.8)
Eswatini	0.3 (0.1-0.4)	0.1 (0.1-0.3)	8.5 (4.2-13.9)	4.2 (2-7.8)
Ethiopia	0 (0-0.1)	0.1 (0-0.2)	1.1 (0.5-2)	2.5 (1.2-5.2)
Fiji	0 (0-0.1)	0 (0-0.1)	2.5 (1.3-4.4)	2 (1.1-3.7)
Finland	0.3 (0.2-0.5)	0.3 (0.2-0.5)	8.7 (5.5-11.9)	8.2 (5.6-11.8)
France	0.2 (0.1-0.2)	0.1 (0.1-0.1)	5.6 (3.6-8.6)	3.4 (2.2-5.5)
Gabon	0.1 (0-0.1)	0.3 (0.1-0.5)	2.2 (1-3.8)	7 (3.5-12.6)
Gambia	0.2 (0.1-0.4)	0.2 (0.1-0.4)	5.3 (2.5-9.3)	5 (2.2-9.9)
Georgia	0.1 (0-0.1)	0.3 (0.2-0.4)	2.6 (1.7-3.8)	8 (6.1-10.7)
Germany	0.4 (0.3-0.6)	0.4 (0.3-0.6)	12.7 (8.4-17.3)	11.7 (8.3-16.3)
Ghana	0.2 (0.1-0.4)	0.2 (0.1-0.4)	4.5 (2.1-8.3)	4.4 (2-8.3)
Greece	0.1 (0.1-0.2)	0.1 (0.1-0.2)	5.2 (3.1-8.1)	5.1 (3.1-8.2)
Greenland	1.3 (0.7-2.1)	0.8 (0.5-1.5)	28.3 (16.7-42.1)	18.8 (10.9-31.2)
Grenada	0.2 (0.1-0.3)	0.2 (0.1-0.3)	4.8 (2.7-7.3)	4.7 (2.8-7.8)
Guam	0 (0-0)	0.2 (0.2-0.3)	0.9 (0.4-1.9)	8.5 (6.2-11.5)
Guatemala	0 (0-0)	0 (0-0)	1.8 (0.8-3.5)	1 (0.4-2.3)
Guinea	0.2 (0.1-0.4)	0.3 (0.1-0.5)	5.5 (2.3-10.6)	6.1 (2.6-11.7)
Guinea-Bissau	0 (0-0.1)	0.1 (0.1-0.3)	1.2 (0.5-2.2)	3.2 (1.4-6.3)

Guyana	0.1 (0.1-0.2)	0.1 (0.1-0.2)	3.8 (2.3-5.6)	4 (2.6-6.2)
Haiti	0.1 (0-0.2)	0.1 (0-0.2)	3 (1.5-4.9)	3.1 (1.5-5.6)
Honduras	0.1 (0-0.1)	0 (0-0.1)	3.2 (1.7-5.2)	1.7 (0.9-3)
Hungary	2 (1.5-2.6)	0.7 (0.5-0.8)	48.9 (37.8-60.9)	17.7 (13.3-22.2)
Iceland	0.1 (0.1-0.2)	0.1 (0-0.1)	5.1 (3-8.2)	2.8 (1.5-5.2)
India	0.1 (0-0.1)	0.1 (0.1-0.1)	3.5 (1.8-5.9)	3.4 (1.9-5.5)
Indonesia	0 (0-0)	0.1 (0-0.1)	2 (1-3.6)	2.9 (1.5-4.6)
Iran (Islamic Republic of)	0 (0-0)	0 (0-0)	1.6 (0.9-2.7)	1.5 (0.8-2.8)
Iraq	0 (0-0)	0 (0-0.1)	2.1 (1-3.7)	2.5 (1.3-4.3)
Ireland	1.2 (0.9-1.6)	0.2 (0.2-0.3)	26.1 (19.3-33.5)	6.1 (4.2-8.8)
Israel	0.4 (0.2-0.6)	0.2 (0.1-0.3)	10.7 (6.7-14.9)	6.2 (4.1-9.2)
Italy	0.6 (0.4-0.8)	0.2 (0.1-0.3)	14 (10.5-18.3)	5.7 (3.9-8.5)
Jamaica	0.5 (0.3-0.7)	0.3 (0.2-0.5)	11.7 (7.4-17)	8.9 (5.4-13.5)
Japan	0.1 (0.1-0.1)	0.1 (0-0.1)	2.7 (1.8-4.1)	2 (1.3-3)
Jordan	0 (0-0)	0 (0-0)	2.4 (1.2-4.2)	2.3 (1.1-4.5)
Kazakhstan	0.1 (0-0.1)	0 (0-0.1)	2.3 (1.4-3.2)	2.1 (1.4-3.2)
Kenya	0.2 (0.1-0.4)	0.2 (0.1-0.4)	4.4 (2.2-8)	4.6 (2.2-8.6)
Kiribati	0 (0-0.1)	0.1 (0-0.2)	3.4 (1.7-5.8)	5.1 (3-8.3)
Kuwait	0.1 (0-0.1)	0 (0-0)	2.7 (1.5-4.4)	1.3 (0.7-2.4)
Kyrgyzstan	0.1 (0.1-0.1)	0.1 (0.1-0.1)	2.9 (2-3.9)	2.9 (2-4)
Lao People's Democratic Republic	0 (0-0)	0.1 (0-0.1)	2.5 (1.3-4.2)	3.1 (1.9-5.5)
Latvia	0.6 (0.4-0.8)	0.8 (0.6-1.1)	17.2 (12.5-22.4)	20.8 (14.9-27.3)
Lebanon	0.1 (0-0.1)	0.1 (0.1-0.2)	3.1 (1.8-5.4)	5.1 (3.2-8)
Lesotho	0.2 (0.1-0.4)	0.2 (0.1-0.4)	6.5 (3-11.7)	6.5 (3.1-11.4)
Liberia	0.2 (0.1-0.4)	0.2 (0.1-0.4)	5.2 (2.6-8.7)	6.2 (2.7-11.2)
Libya	0.1 (0-0.1)	0.1 (0-0.1)	2.8 (1.6-4.4)	2.8 (1.6-4.7)
Lithuania	0.6 (0.4-0.8)	0.4 (0.3-0.6)	16.6 (11.4-21.9)	11.4 (8.4-14.6)
Luxembourg	0.2 (0.2-0.4)	0.1 (0.1-0.2)	7.4 (4.6-10.5)	4.5 (2.7-7.9)
Madagascar	0.3 (0.1-0.5)	0.2 (0.1-0.4)	5.8 (2.8-10.4)	4.1 (1.8-8.2)
Malawi	0.2 (0.1-0.5)	0.5 (0.2-1)	5.5 (2.6-9.7)	10.3 (4.8-19.1)
Malaysia	0 (0-0)	0 (0-0)	2 (1-3.7)	1.7 (1-3.1)
Maldives	0 (0-0)	0 (0-0)	2.5 (1.1-4.7)	1.9 (0.8-3.6)
Mali	0.1 (0-0.1)	0.2 (0.1-0.3)	1.9 (0.8-3.5)	4.3 (1.9-8.3)
Malta	0.5 (0.3-0.8)	0.2 (0.1-0.3)	12.8 (8.5-17.6)	5.9 (3.7-9.1)
Marshall Islands	0.1 (0-0.2)	0.2 (0.1-0.4)	4 (2.2-6.5)	7.9 (4.2-13.6)
Mauritania	0.1 (0.1-0.3)	0.2 (0.1-0.4)	3.5 (1.7-6.6)	4.8 (2.1-9.7)
Mauritius	0.1 (0-0.1)	0.1 (0.1-0.1)	3 (1.9-4.6)	3.8 (2.6-5.6)
Mexico	0.2 (0.1-0.3)	0.1 (0-0.1)	6.4 (3.8-9.9)	2.6 (1.5-4.4)
Micronesia (Federated States of)	1 (0.5-1.6)	1 (0.5-1.6)	25.5 (13.8-41.3)	25.6 (15.4-40.3)
Monaco	0.5 (0.2-0.8)	0.2 (0.1-0.5)	11.8 (6.8-18.1)	6.2 (2.7-11.6)
Mongolia	0 (0-0)	0 (0-0)	1.3 (0.6-2.4)	1.4 (0.7-2.7)
Montenegro	0.1 (0.1-0.2)	0.1 (0.1-0.1)	4.3 (2.7-6.6)	4 (2.4-6.3)

Morocco	0 (0-0.1)	0 (0-0.1)	1.5 (0.8-2.6)	1.4 (0.7-2.4)
Mozambique	0.1 (0-0.1)	0.1 (0.1-0.3)	1.8 (0.8-3.2)	3.4 (1.6-6.2)
Myanmar	0 (0-0.1)	0.1 (0-0.1)	3.8 (1.8-6.6)	2.5 (1.4-4.2)
Namibia	0.2 (0.1-0.4)	0.3 (0.1-0.5)	8 (4.2-13.1)	8.3 (4.4-13.7)
Nauru	0.1 (0.1-0.2)	0.3 (0.1-0.4)	5.6 (3.2-8.6)	9.8 (5.8-15.7)
Nepal	0.1 (0-0.1)	0.1 (0-0.1)	5.9 (2.7-9.9)	4.9 (2.6-9)
Netherlands	0.8 (0.6-1.1)	0.5 (0.3-0.6)	19.6 (14.6-26.4)	11.1 (7.7-15.2)
New Zealand	0.5 (0.4-0.7)	0.2 (0.1-0.3)	10.6 (7.6-14.2)	3.7 (2.5-5.3)
Nicaragua	0 (0-0)	0 (0-0)	1.6 (0.7-3.1)	1.2 (0.5-2.2)
Niger	0.1 (0-0.1)	0.1 (0-0.3)	1.4 (0.5-2.9)	3.2 (1.3-6.9)
Nigeria	0.2 (0.1-0.3)	0.1 (0-0.2)	3.7 (1.7-7)	3 (1.2-5.7)
Niue	0.1 (0-0.1)	0.1 (0-0.2)	2.7 (1.6-4.5)	3.7 (2.1-6.4)
North Macedonia	0.1 (0.1-0.1)	0.1 (0-0.1)	3.8 (2.2-6.2)	3.5 (2.1-5.8)
Northern Mariana Islands	0 (0-0)	0.3 (0.1-0.4)	1.6 (0.7-2.9)	8 (4.7-12.7)
Norway	0.7 (0.4-0.9)	0.1 (0.1-0.1)	15.4 (10.7-20.8)	3.4 (2.2-5.6)
Oman	0.1 (0-0.1)	0.1 (0-0.1)	3.2 (1.9-5.1)	2.5 (1.4-4.2)
Pakistan	0.1 (0-0.1)	0.1 (0-0.2)	4.9 (2.5-8.3)	4.3 (2.2-7.4)
Palau	0.1 (0-0.1)	0.1 (0.1-0.2)	2.9 (1.7-4.9)	4.1 (2.3-6.8)
Palestine	0 (0-0.1)	0 (0-0.1)	2.5 (1.4-4.5)	2.5 (1.4-4.1)
Panama	0.3 (0.2-0.4)	0.1 (0-0.1)	6.8 (3.8-10.6)	2 (1.1-3.5)
Papua New Guinea	0.1 (0-0.1)	0.1 (0-0.2)	2.7 (1.5-4.5)	3.6 (2-6.2)
Paraguay	0.1 (0.1-0.2)	0.1 (0.1-0.2)	5.2 (3-8.5)	4.5 (2.6-7.1)
Peru	0 (0-0)	0 (0-0)	1 (0.4-1.9)	1.1 (0.5-2)
Philippines	0.1 (0-0.1)	0.1 (0.1-0.1)	4.4 (2.7-7.5)	3.4 (2.1-5.4)
Poland	1.3 (1-1.7)	0.6 (0.4-0.8)	32.9 (24.3-40.7)	14.6 (10.7-19.3)
Portugal	0.5 (0.4-0.7)	0.2 (0.2-0.3)	12.6 (9.1-16.5)	6.6 (4.7-8.6)
Puerto Rico	0.2 (0.1-0.3)	0.2 (0.1-0.3)	5.8 (3.6-8.4)	5.3 (3.4-7.7)
Qatar	0.1 (0.1-0.2)	0 (0-0.1)	3.6 (2-5.9)	1.8 (1-3)
Republic of Korea	0.1 (0.1-0.1)	0 (0-0.1)	3 (1.9-4.3)	1.5 (1-2.4)
Republic of Moldova	0.3 (0.2-0.4)	0.4 (0.3-0.6)	7.3 (5-9.6)	11.6 (8.3-15.8)
Romania	0.4 (0.3-0.6)	0.3 (0.2-0.4)	12 (8.3-15.7)	8.1 (6.1-10.5)
Russian Federation	1 (0.7-1.4)	0.9 (0.7-1.3)	27.4 (19.7-36.7)	23.2 (16.8-31.2)
Rwanda	0.3 (0.1-0.5)	0.6 (0.3-1.2)	7.1 (3.6-11.9)	13.2 (7-24.6)
Saint Kitts and Nevis	0.2 (0.1-0.4)	0.2 (0.1-0.3)	6.1 (3.3-9.6)	4.8 (2.6-8.3)
Saint Lucia	0.4 (0.2-0.6)	0.3 (0.2-0.4)	9.8 (5.8-14.7)	7.6 (4.8-11.4)
Saint Vincent and the Grenadines	0.1 (0-0.1)	0.1 (0.1-0.1)	2.1 (1.2-3.5)	3.2 (1.9-5.1)
Samoa	0.1 (0-0.1)	0.2 (0.1-0.3)	4.5 (2.5-7.5)	7.1 (4-11.5)
San Marino	0.2 (0.1-0.4)	0.1 (0.1-0.2)	6.8 (3.8-10.8)	3.7 (2-6.3)
Sao Tome and Principe	0.1 (0-0.1)	0.2 (0.1-0.4)	1.7 (0.8-2.9)	4.6 (2.1-9.1)
Saudi Arabia	0 (0-0)	0 (0-0)	0.9 (0.4-1.6)	1 (0.5-1.8)
Senegal	0.1 (0-0.2)	0.1 (0-0.2)	3.5 (1.6-6.1)	2.9 (1.3-5.7)
Serbia	0.2 (0.1-0.3)	0.2 (0.1-0.3)	6.3 (4-9.2)	6 (3.9-8.3)
Seychelles	0 (0-0.1)	0 (0-0.1)	2.3 (1.3-4)	2.3 (1.3-3.8)

Sierra Leone	0.2 (0.1-0.5)	0.3 (0.1-0.5)	6.5 (3-11.9)	7 (3.3-13.1)
Singapore	0.1 (0.1-0.2)	0 (0-0)	3.5 (2.2-5.1)	1 (0.6-1.6)
Slovakia	0.3 (0.2-0.4)	0.3 (0.2-0.5)	8 (5.1-11)	8.7 (6.1-12.1)
Slovenia	0.3 (0.2-0.4)	0.1 (0.1-0.2)	8.2 (5.4-11.4)	4 (2.8-5.8)
Solomon Islands	0.1 (0-0.2)	0.2 (0.1-0.3)	4.1 (2.3-6.5)	6.7 (4-10.5)
Somalia	0 (0-0.1)	0.1 (0-0.2)	1.4 (0.6-2.8)	2.1 (0.9-4.2)
South Africa	0.6 (0.4-0.9)	0.5 (0.3-0.7)	17.1 (10.6-25.7)	13.5 (9.3-18.9)
South Sudan	0.1 (0-0.2)	0.1 (0.1-0.3)	2.3 (1-4.7)	3.5 (1.5-7.2)
Spain	0.5 (0.3-0.6)	0.2 (0.2-0.3)	11.1 (8-14.8)	6.9 (4.7-9.8)
Sri Lanka	0 (0-0)	0 (0-0)	2 (1-3.4)	1 (0.5-1.8)
Sudan	0 (0-0)	0 (0-0)	1.1 (0.5-1.9)	1 (0.5-1.9)
Suriname	0.1 (0-0.1)	0.1 (0-0.1)	3.6 (2-6)	3.1 (1.7-4.9)
Sweden	0.5 (0.3-0.7)	0.2 (0.1-0.2)	10.7 (7.3-15)	5.2 (3.2-7.7)
Switzerland	0.5 (0.3-0.7)	0.2 (0.1-0.3)	12.3 (8-16.7)	6.1 (4-9.6)
Syrian Arab Republic	0.1 (0-0.1)	0 (0-0.1)	3.1 (1.7-5)	2.2 (1.2-3.8)
Taiwan (Province of China)	0 (0-0)	0 (0-0)	1.6 (0.7-2.9)	1 (0.5-1.9)
Tajikistan	0 (0-0.1)	0 (0-0.1)	2 (1.2-3.1)	1.2 (0.6-2)
Thailand	0 (0-0)	0 (0-0)	2 (0.9-3.5)	1.4 (0.7-2.8)
Timor-Leste	0 (0-0)	0 (0-0.1)	2.1 (1-3.6)	2.1 (1.1-3.7)
Togo	0.3 (0.1-0.5)	0.2 (0.1-0.4)	7.1 (3.5-12.2)	4.7 (2.3-8.9)
Tokelau	0.1 (0-0.1)	0.1 (0-0.2)	3.2 (1.8-5.6)	4.2 (2.5-6.7)
Tonga	0.1 (0.1-0.2)	0.2 (0.1-0.4)	5.4 (3.3-8.3)	7.9 (4.3-12.3)
Trinidad and Tobago	0.3 (0.2-0.5)	0.3 (0.2-0.5)	8.5 (5.2-12.2)	7.5 (4.5-11.9)
Tunisia	0.1 (0-0.1)	0 (0-0.1)	2.7 (1.5-4.5)	2.5 (1.4-4)
Turkey	0.3 (0.2-0.4)	0.2 (0.1-0.3)	8.5 (5-13.1)	5.9 (4-8.4)
Turkmenistan	0.1 (0-0.1)	0.1 (0-0.1)	2.3 (1.4-3.3)	2 (1.1-3)
Tuvalu	0.1 (0-0.1)	0.2 (0.1-0.4)	3.6 (2.1-5.6)	8.2 (4.5-13.4)
Uganda	0.1 (0-0.2)	0.2 (0.1-0.4)	2.6 (1.2-4.7)	4.2 (2.2-7.7)
Ukraine	0.9 (0.6-1.3)	0.6 (0.5-0.8)	21 (13.8-28.6)	15.6 (11.4-19.9)
United Arab Emirates	0.1 (0-0.1)	0 (0-0.1)	2.6 (1.1-4.9)	1.6 (0.8-2.8)
United Kingdom	1 (0.7-1.3)	0.3 (0.2-0.5)	21.5 (15.4-27.4)	8.4 (5.7-11.6)
United Republic of Tanzania	0.2 (0.1-0.4)	0.3 (0.1-0.5)	5.5 (2.9-9.2)	7.5 (3.7-13)
United States of America	0.4 (0.3-0.6)	0.4 (0.3-0.5)	12 (8.8-15.9)	10.8 (7.8-14.2)
United States Virgin Islands	0.2 (0.1-0.4)	0.2 (0.1-0.2)	5.7 (3.3-8.9)	4.8 (2.9-7.3)
Uruguay	0 (0-0)	0 (0-0)	1.3 (0.6-2.3)	1.3 (0.6-2.6)
Uzbekistan	0 (0-0)	0 (0-0)	0.7 (0.3-1.3)	1 (0.5-1.6)
Vanuatu	0.1 (0-0.1)	0.1 (0-0.1)	2.8 (1.6-4.5)	3.2 (1.9-5.4)
Venezuela (Bolivarian Republic of)	0.1 (0.1-0.2)	0 (0-0.1)	4.6 (2.6-7.4)	2 (0.9-3.9)
Viet Nam	0 (0-0)	0.1 (0-0.1)	2.2 (1.1-4)	2.5 (1.5-3.9)
Yemen	0.1 (0-0.1)	0 (0-0.1)	2.9 (1.5-4.7)	2.3 (1.2-4.1)
Zambia	0.3 (0.1-0.6)	0.3 (0.2-0.7)	7.1 (3.5-12.2)	8.5 (4.2-16)

Zimbabwe	0 (0-0)	0 (0-0)	1.9 (0.9-3.7)	1.1 (0.5-2.4)

Note: Data in parentheses represent the 95% uncertainty intervals (UIs). Rates are reported per 100,000 population.

Abbreviations: ASMR, age-standardized mortality rate; ASDR, age-standardized disability-adjusted life years rate; UI, uncertainty interval.

2. **Supplementary Figure 1.** The association between the disability-adjusted life years (DALYs) burden of lower extremity peripheral arterial disease attributable to smoking and socio-demographic index (SDI) from 1990 to 2021.

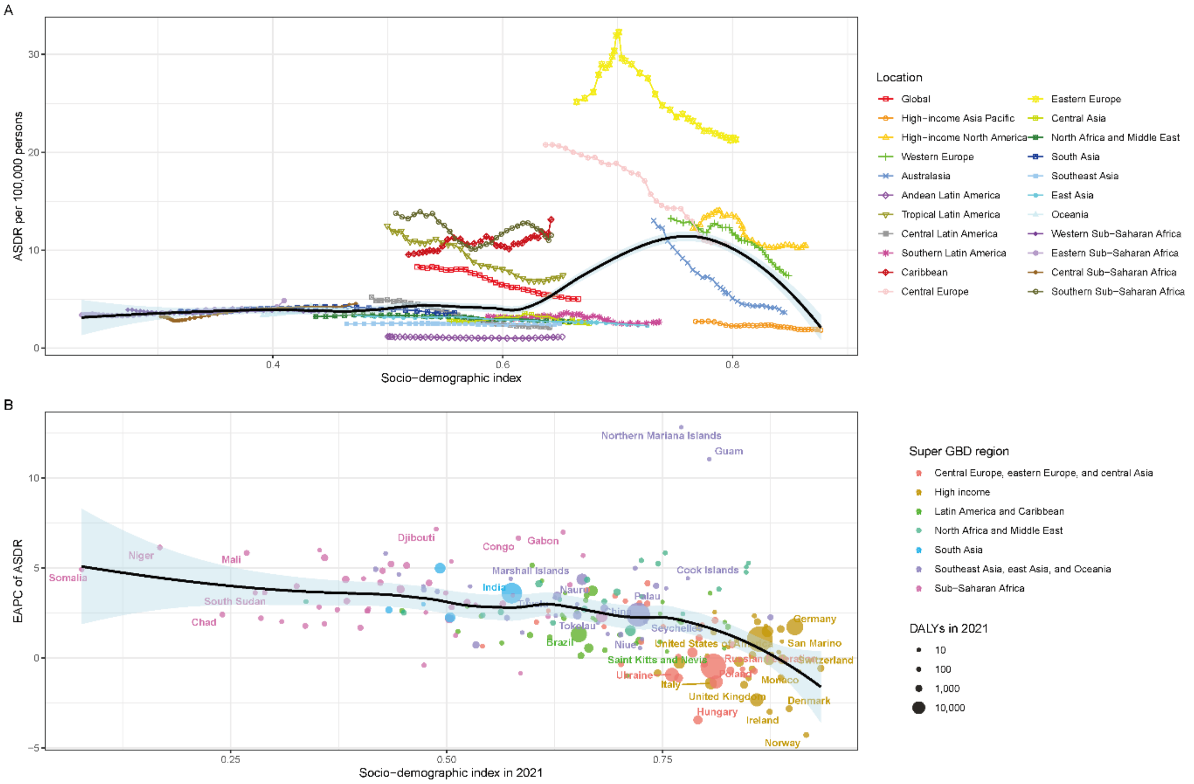

(A) The trends in age-standardized DALY rate (ASDR) across 21 Global Burden of Disease (GBD) regions from 1990 to 2021. Colored lines represent the trajectories of different regions over time. The black solid line represents the expected ASDR based on SDI values across all regions.

(B) The correlation between the estimated annual percentage change (EAPC) of ASDR (1990–2021) and the SDI in 2021 across 204 countries and territories. Circles represent countries or territories, with the size proportional to the absolute number of DALYs in 2021. The colors of the circles denote the seven GBD super-regions. The black line represents the polynomial regression fitting curve, and the blue shaded area indicates the 95% confidence interval.

Abbreviations: ASDR, age-standardized DALY rate; DALYs, disability-adjusted life years; EAPC, estimated annual percentage change; SDI, socio-demographic index; GBD, Global Burden of Disease.

=

3. **Supplementary Figure 2.** Joinpoint regression analysis of global trends in the burden of lower extremity peripheral arterial disease attributable to smoking from 1990 to 2021.

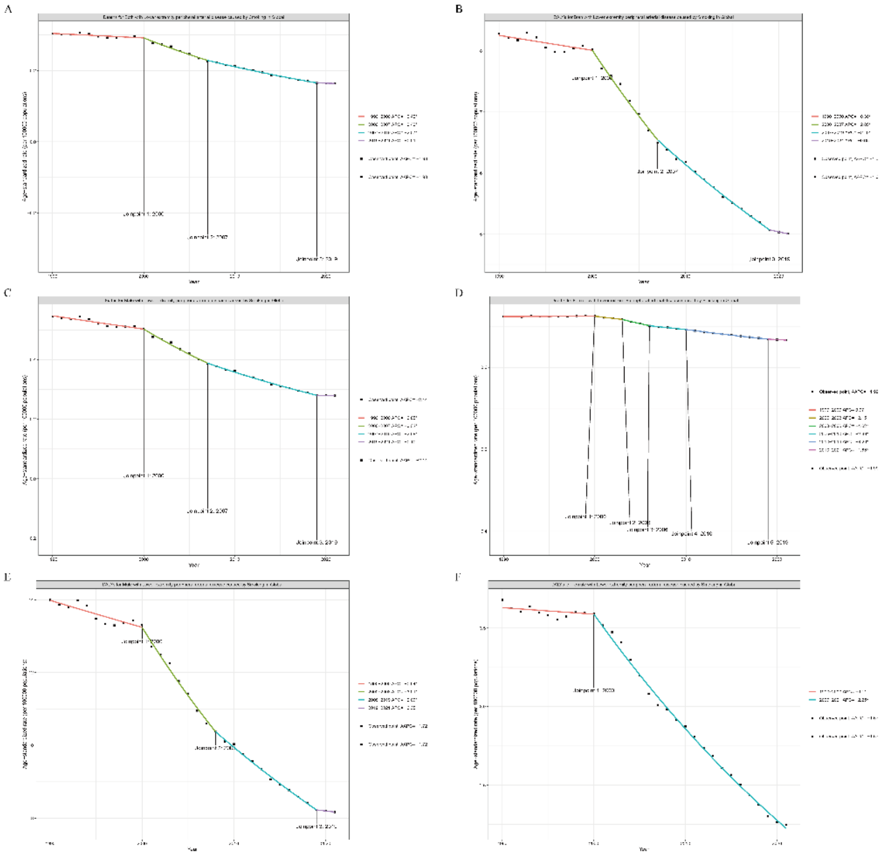

(A)Age-standardized mortality rate (ASMR) for both sexes;(B) Age-standardized DALY rate (ASDR) for both sexes;(C) ASMR for males;(D) ASMR for females;(E) ASDR for males;(F) ASDR for females.

Notes: The black dots represent the observed age-standardized rates. The colored lines represent the trends fitted by the Joinpoint regression model. APC indicates the Annual Percentage Change for each segment, and AAPC indicates the Average Annual Percentage Change over the entire study period. An asterisk (*) denotes a statistically significant trend ($P < 0.05$).

Abbreviations: ASMR, age-standardized mortality rate; ASDR, age-standardized disability-adjusted life years rate; APC, annual percentage change; AAPC, average annual percentage change.

4. **Supplementary Figure 3.** The age-specific proportion and rate changes in the burden of lower extremity peripheral arterial disease attributable to smoking from 1990 to 2021.

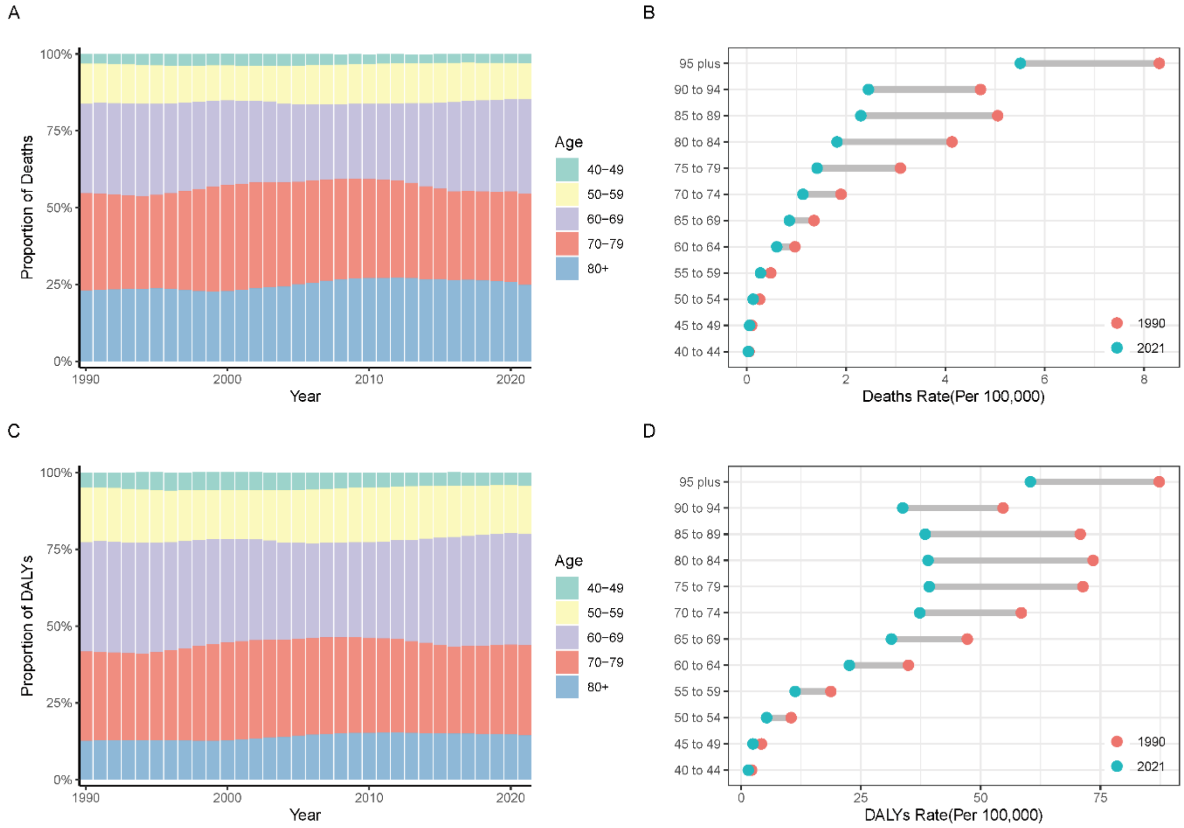

(A, C) Temporal trends in the proportional contribution of different age groups to total deaths (A) and DALYs (C) from 1990 to 2021. The stacked bar charts display the distribution across five age categories: 40-49, 50-59, 60-69, 70-79, and 80+ years.
(B, D) Changes in age-specific death rates (B) and DALY rates (D) (per 100,000 population) between 1990 and 2021. The red circles represent the rates in 1990, and the blue circles represent the rates in 2021. The gray lines connect the two time points for each age group, illustrating the magnitude and direction of the change.
Abbreviations: DALYs, disability-adjusted life years.

5. **Supplementary Figure 4.** The age-specific proportion and rate changes in mortality of lower extremity peripheral arterial disease attributable to smoking by sex from 1990 to 2021.

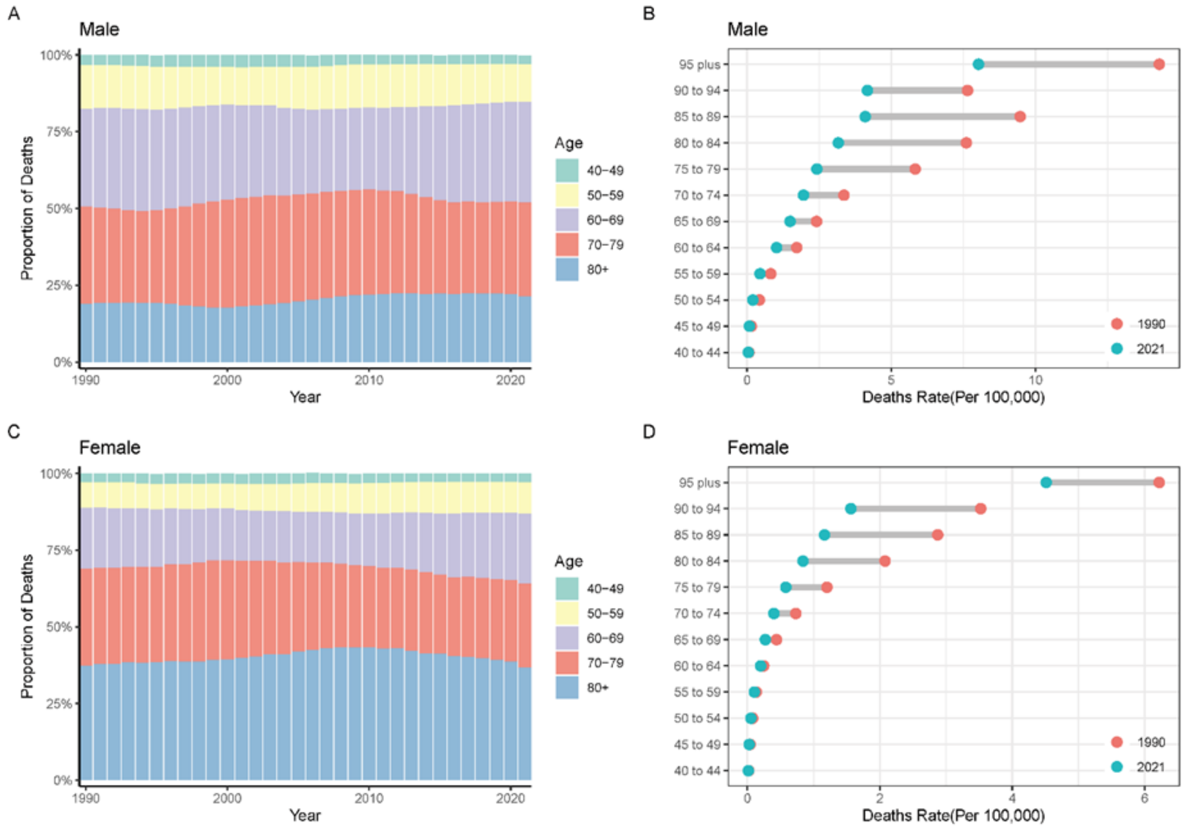

(A) The temporal trends in the relative proportion of deaths across different age groups for males from 1990 to 2021.

(B) Comparison of age-specific mortality rates (per 100,000 population) for males between 1990 and 2021.

(C) The temporal trends in the relative proportion of deaths across different age groups for females from 1990 to 2021.

(D) Comparison of age-specific mortality rates (per 100,000 population) for females between 1990 and 2021.

Notes: In panels B and D, red dots represent the rates in 1990, and blue dots represent the rates in 2021. The gray lines indicate the magnitude of change over the study period.

6. **Supplementary Figure 5.** The age-specific proportion and rate changes in DALYs of lower extremity peripheral arterial disease attributable to smoking by sex from 1990 to 2021

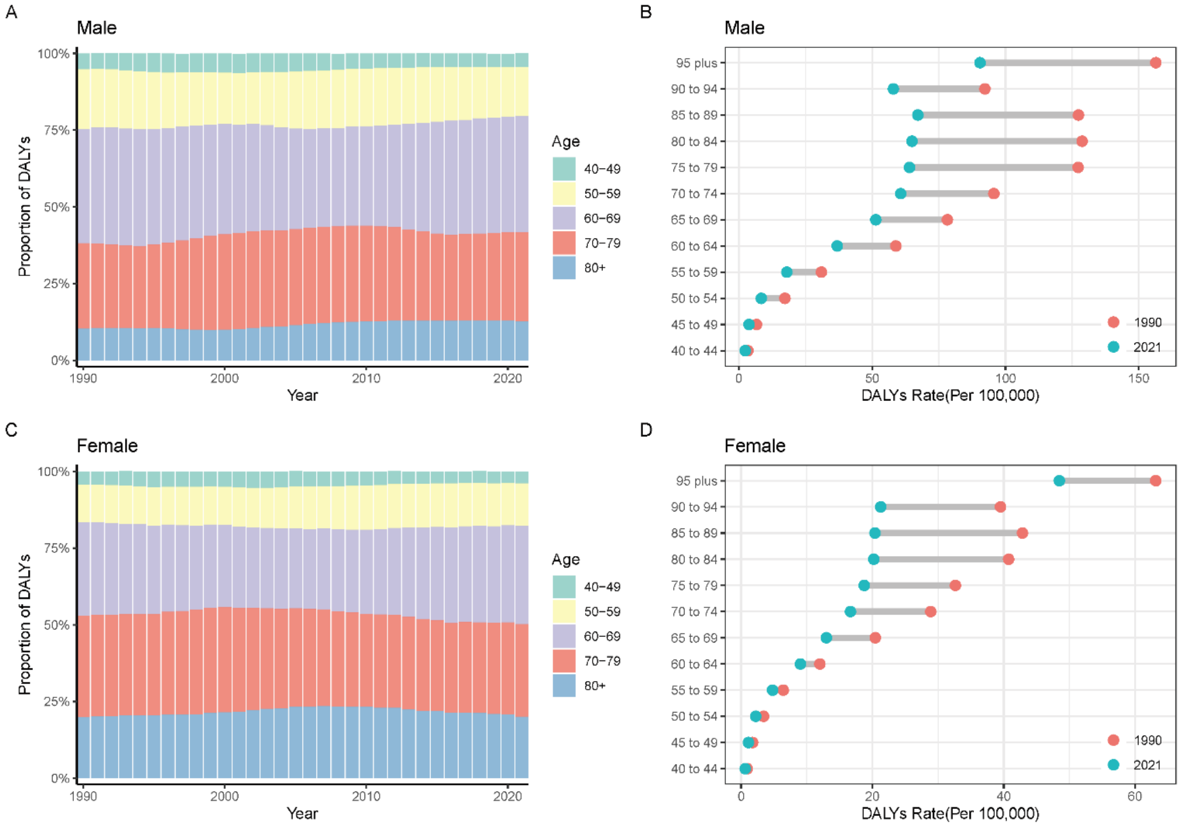

(A) The temporal trends in the relative proportion of DALYs across different age groups for males from 1990 to 2021.

(B) Comparison of age-specific DALY rates (per 100,000 population) for males between 1990 and 2021.

(C) The temporal trends in the relative proportion of DALYs across different age groups for females from 1990 to 2021.

(D) Comparison of age-specific DALY rates (per 100,000 population) for females between 1990 and 2021.

Notes: In panels B and D, red dots represent the rates in 1990, and blue dots represent the rates in 2021. The gray lines indicate the magnitude of change over the study period.

Abbreviations: DALYs, disability-adjusted life years.

7. **Supplementary Figure 6.** The global spatial distribution of the burden of lower extremity peripheral arterial disease attributable to smoking in 1990 and 2021.

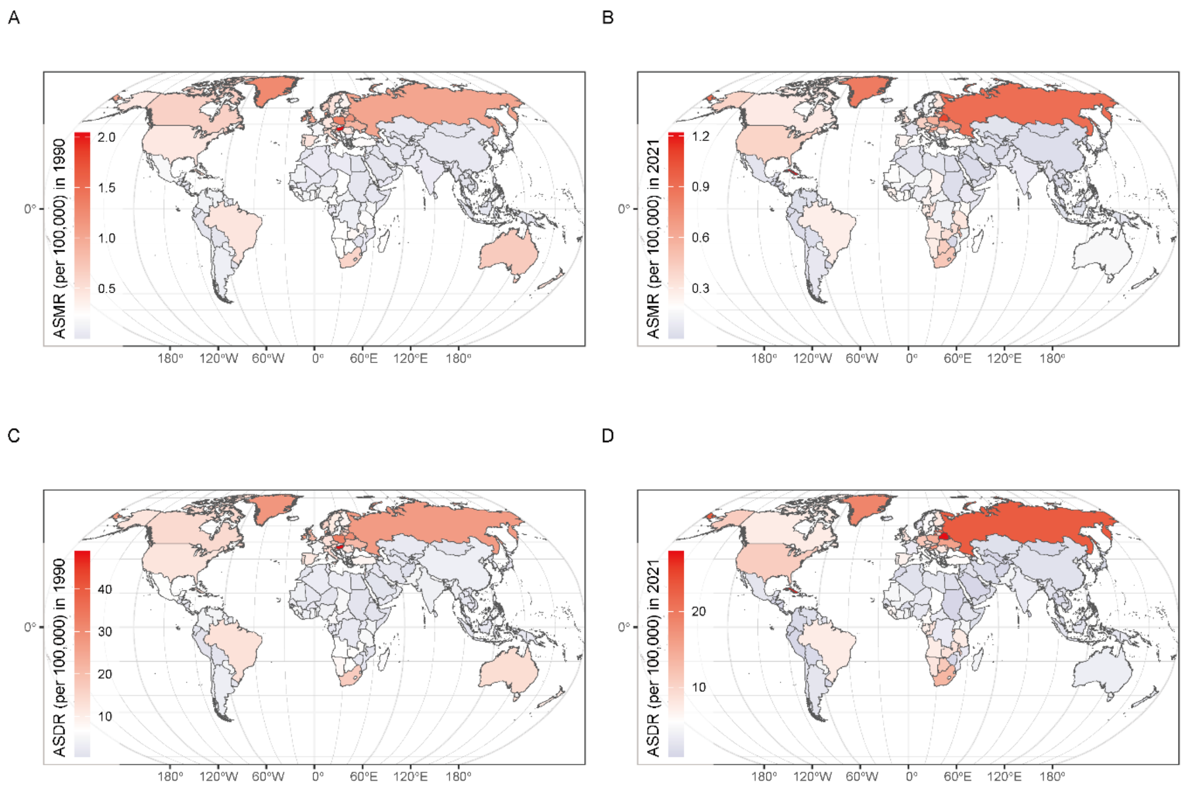

(A)The age-standardized mortality rate (ASMR) in 1990.(B) The age-standardized mortality rate (ASMR) in 2021.(C) The age-standardized DALY rate (ASDR) in 1990.(D) The age-standardized DALY rate (ASDR) in 2021.

Notes: Countries and territories are colored according to the magnitude of the age-standardized rates (per 100,000 population). Red indicates a high burden, while blue indicates a low burden.

Abbreviations: ASMR, age-standardized mortality rate; ASDR, age-standardized disability-adjusted life years rate; DALYs, disability-adjusted life years.

© **2026 Chen Z. et al.**
